# Aspects of images in magnetic resonance of liver tumors treated with transarterial selective internal radiotherapy with yttrium-90

**DOI:** 10.1590/S1679-45082017RC4015

**Published:** 2017-12-08

**Authors:** Breno Boueri Affonso, Joaquim Maurício da Motta-Leal-Filho, Francisco de Assis Cavalcante, Francisco Leonardo Galastri, Rafael Noronha Cavalcante, Priscila Mina Falsarella, Felipe Nasser, Rodrigo Gobbo Garcia

**Affiliations:** 1 Hospital Israelita Albert Einstein, São Paulo, SP, Brazil

**Keywords:** Chemoembolization, therapeutic/methods, Yttrium radioisotopes/therapeutic use, Liver neoplasms/radiotherapy, Magnetic resonance imaging, Case reports, Quimioembolização terapêutica/métodos, Radioisótopos de ítrio/uso terapêutico, Neoplasias hepáticas/radioterapia, Imagem por ressonância magnética, Relatos de casos

## Abstract

Transarterial selective internal radiation therapy with yttrium-90, also known as radioembolization, is a therapy based on the administration of resin or glass microspheres loaded with the radioisotope yttrium-90, via selective arterial catheterization of tumor-feeding vessels. It is classified as a type of locoregional therapy and its main goal is to treat patients with primary or secondary hepatic lesions that are unresectable and not responsive to other therapies. Since it is a new technology still restricted to very few hospitals in Brazil, but used in patients throughout the country, it is necessary to demonstrate the main aspects of hepatic lesions treated with selective internal radiation therapy found in magnetic resonance imaging, and to make specific considerations on interpretation of these images. The objective of this report is to demonstrate the main aspects of magnetic resonance imaging of unresectable primary or secondary hepatic lesions, in patients submitted to transarterial selective internal radiation therapy.

## RESUMO

A radioterapia interna seletiva transarterial com ítrio-90, também conhecida como radioembolização, é uma terapia baseada na administração de microesferas de resina ou vidro carregadas com o radioisótopo ítrio-90, via cateterismo arterial seletivo dos vasos nutridores do tumor. É classificada como um tipo de terapia locorregional e seu principal objetivo é tratar pacientes portadores de lesões hepáticas primárias ou secundárias irressecáveis e não responsivas a outras terapias. Por se tratar de uma nova tecnologia, portanto ainda restrita a pouquíssimos hospitais no Brasil (ainda que utilizada em todo país), é necessário demonstrar os principais aspectos de imagem das lesões hepáticas tratadas com radioterapia interna seletiva transarterial encontrados em exame de ressonância magnética, além de delinear considerações específicas de interpretação destas imagens. O objetivo deste relato é demonstrar os principais aspectos encontrados em ressonância magnética de lesões hepáticas irressecáveis, primárias ou secundárias, de pacientes submetidos à radioterapia interna seletiva transarterial.

## INTRODUCTION

The transarterial selective internal radiation therapy (SIRT) with yttrium-90 is a locoregional therapy based on administration of microspheres loaded with yttrium-90, through selective arterial catheterization of tumor-feeding vessels. The main objective is to treat patients with primary or secondary hepatic lesions that are unresectable and do not respond to other therapies.^(^ ^1^ ^,^ ^2^ ^)^

By passing the catheter through the common femoral artery, and using a coaxial catheter system, the selective catheterization of the celiac trunk and of the hepatic artery is performed; then, the microparticle set (yttrium −90) is selectively delivered to the hepatic arterial circulation (left, right or both), which feeds the neoplastic region. The microspheres are permanent implant (they are not absorbed) and used only once in each patient. Their mean diameter is 32.5 *μ* (20 to 60 *μ* )^(^ ^3^ ^,^ ^4^ ^)^ and the microspheres do not cause an embolic effect, that is, there is no occlusion of the tumor-feeding vessels. Yttrium-90 is a high-energy radioisotope that emits beta radiation with a maximum reach of 11mm (mean penetration: 2.5mm) and a half-life of 64.1 hours.^(^ ^5^ ^)^

The procedure is performed by a team of interventional radiologists. However, a successful treatment demands involving a multidisciplinary team, comprising professionals from Diagnostic Radiology, Nuclear Medicine and Oncology, aiming to properly select the patients, check tumor staging, look for liver to lung shunt, calculate the yttrium-90 dose, and follow-up the treated lesions.^(16)^

It is a new technology available at three hospitals in Brazil, but used in patients from throughout the country. It is important to demonstrate the main aspects of images of hepatic lesions treated with SIRT and found in magnetic resonance imaging (MRI), and make specific considerations on interpretation of these images. Therefore, this report aims to contribute to expanding knowledge in Brazilian Radiology.

The purpose of this study was to demonstrate the main aspects found in MRI of primary or secondary unresectable hepatic lesions treated with SIRT.

All patients signed an Informed Consent before the procedure and follow-up exams. Our organization does not require approval by the Ethics Committee for case reports.

We used MR images of patients treated by the SIRT technique at a quaternary care hospital, from November 2014 to July 2016. A total of 35 SIRT/yttrium-90 procedures were performed, and four cases were selected for presenting characteristic MR images found in this therapy.

The images were acquired in 3T MRI devices (MAGNETOM Prisma 3T, Siemens, Berlin, Germany) and 1.5T (Signa HDxt 1.5T, GE Healthcare, Milwaukee, United States). The protocol used to assess the hepatic lesions included the sequences: coronal T2, axial diffusion, axial T2 (TE 80), axial T2 (TE 180), axial T1 gradient-echo (in phase/out of phase), fat saturation axial T1 before and after (dynamic arterial, portal and late phases), and post-contrast fat saturation axial T1. Two control exams were performed, two and six months after the procedure.

## CASE REPORTS

### Case 1

A 56-year-old male patient, diagnosed as pancreatic adenocarcinoma in March 2014, associated with multinodular hepatic metastasis. He initiated on chemotherapy with FOLFIRINOX for four months (discontinued due to systemic toxicity), changed to chemotherapy with gemcitabine, for 4 more months. He presented partial response of the pancreatic tumor, progression of hepatic lesions, and worsening of tumor markers (CA19.9 from 2,100 to 18,000U/mL).

**Figure 1:**
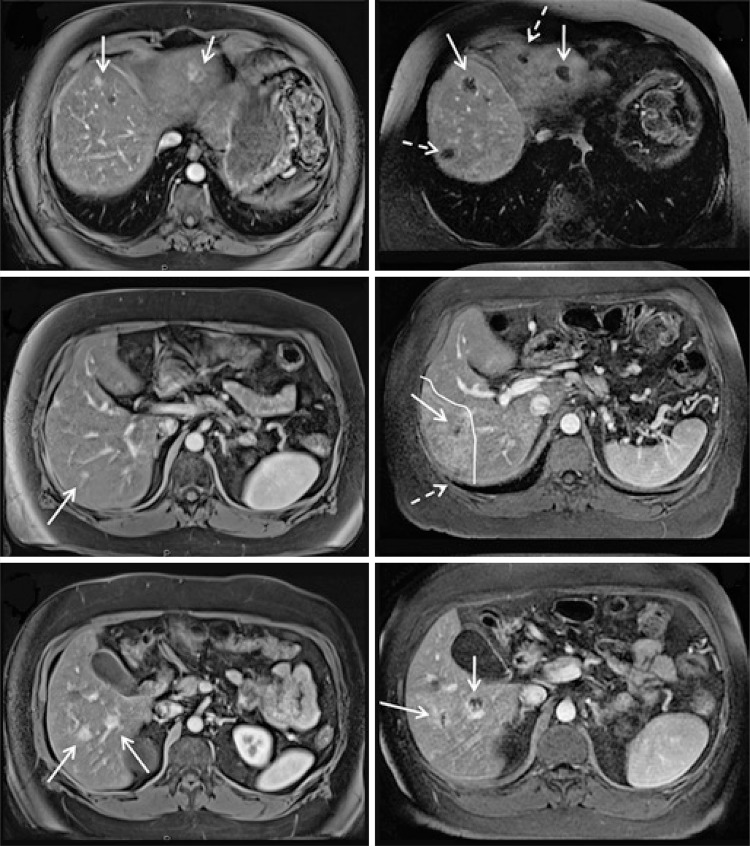
Upper abdomen magnetic resonance, T1-weighted with fat saturation after contrast, in portal phase, axial section, showing secondary hepatic lesions in (A) segments II and IVa (white arrows), (B) segment VII (white arrow) and (C) segment VI (white arrows). Upper abdomen magnetic resonance, T1-weighted with fat saturation after contrast, in portal phase, axial section, showing secondary hepatic lesions in (D) segments II and IVa (white arrows), (E) segment VII (white arrow) and (F) segment VI (white arrows). There are necrotic areas, which showed no lesions in a previous study: images D and E (dotted white arrows) and image E, area of hyperenhancement in segment VII (white arrow)

Abdominal MRI after second-line systemic chemotherapy showed growth of secondary hepatic lesions in segments II, IVa ( Figure 1 A), VII ( Figure 1 B) and VI ( Figure 1 C), despite the chemotherapeutic management. In an Oncology multidisciplinary meeting, it was decided to perform SIRT.

Three months after SIRT, there was virtually complete necrosis of the lesions observed in the previous MRI ( Figure 1 D to 1 F). Moreover, there were necrotic areas not showing the previous lesions ( Figure 1 D to 1 E). These findings may correspond to necrosis of recent secondary lesions or from the hepatic parenchyma. A wedge-shaped hyperenhancement area stands out around the lesion in segment VII ( Figure 1 E).

### Case 2

A 66-year-old female patient, diagnosed as multiple hepatic metastases of an adenocarcinoma of unknown primary site, according to percutaneous hepatic biopsy. Past history of breast cancer appropriately treated six years ago.

**Figure 2:**
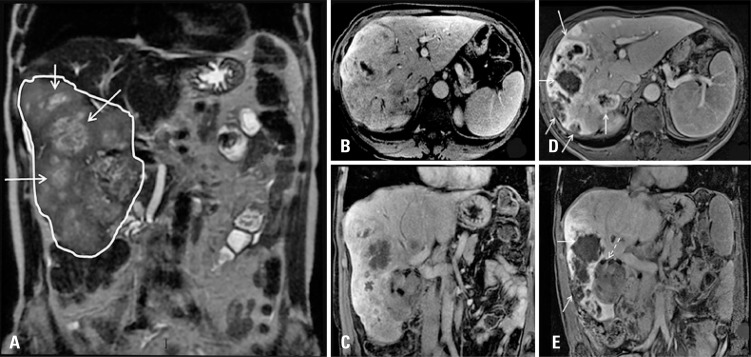
(A) Magnetic resonance imaging of upper abdomen in T2, with no fat saturation, in coronal section, showing bulky hepatic tumor (metastases) located preferably in the right hepatic lobe (white line). Diffuse heterogeneity of coalescent lesions, with high signal central areas (white arrows). (B-E) Magnetic resonance imaging of upper abdomen in T1, with fat saturation, after contrast, in the axial and coronal sections: in B and C, before SIRT, note the bulky hepatic tumor (metastases) located, preferably in the right lobe. In D and E, 2 months after SIRT, there are extensive necrotic areas of infiltrating lesions in the right hepatic lobe (white arrows). Complete necrosis of the tumor lesion in the gallbladder bed stand out (dotted white arrow)

An abdominal MRI showed a bulky hepatic tumor occupying preferably the right hepatic lobe (white line), not involving the portal vein, but it was unresectable ( Figure 2 A). There was diffuse heterogeneity of the coalescent lesions, with central areas suggesting necrosis ( Figure 2 A). Despite the bulky tumor mass, it did not affect over 60% of total hepatic volume, thus allowing performance of SIRT.

Magnetic resonance imaging was performed after 60 days and revealed extensive necrotic areas of infiltrating lesions in the right hepatic lobe, besides complete necrosis of the lesion in the gallbladder bed ( Figure 2 B and 2 E).

### Case 3

A 57-year-old male patient was diagnosed, in 2012, as urothelial cell carcinoma in the left kidney, with lymph node involvement, after presenting macroscopic hematuria. He was submitted to nephroureterectomy followed by systemic chemotherapy with MVAC protocol (methotrexate, vinblastine, doxorubicin and cisplatin) during 6 months. He remained with no evidence of the disease for 22 months.

**Figure 3:**
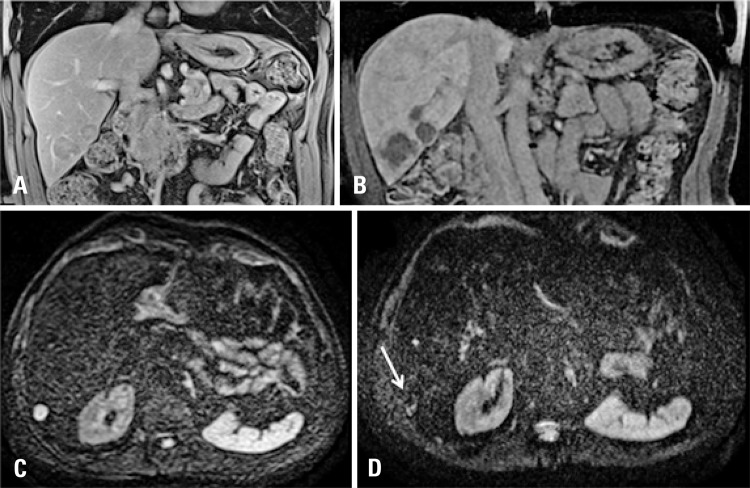
Upper abdomen magnetic resonance, T1-weighted with fat saturation after contrast, in portal phase, coronal section. (A) Two viable nodules in segment VI of the right hepatic lobe (arrows). (B) Complete necrosis of tumor lesions in segment VI (arrows). Larger lesions (inflammation) were observed, but some exuberant necrotic areas appeared (C and D). Upper abdomen image, axial section, diffusion sequence, shows, (C) before SIRT, a hepatic lesion in segment VI with diffusion restriction (arrow), confirmed in the apparent diffusion coefficient map (not represented here), indicating a high-cellularity neoplastic lesion (viable tumor). In D, after SIRT, the diffusion restriction image disappeared in the same position previously described (white arrow)

In March 2015, the MRI showed a single liver metastasis. Stereotactic body radiotherapy (SBRT) was performed, and he underwent four chemotherapy cycles of gemcitabine and carboplatin, remaining with no evidence of disease for 6 months. In December 2015, in a control MRI, multinodular liver metastases were observed ( Figure 3 A), besides involvement of retroperitoneal lymph nodes. In a multidisciplinary Oncology meeting, it was decided to use the MVAC protocol and perform SIRT for the hepatic disease.

Two months after SIRT, the MRI showed larger tumor lesions and exuberant necrotic areas ( Figure 3 B). The magnetic resonance images in diffusion sequence are excellent to assess tumor viability (displaying restriction to pretreatment diffusion “viable tumor”- ( Figure 3 C). In this case, diffusion restriction was absent (absence of tumor) in the post-treatment with SIRT ( Figure 3 D).

### Case 4

**Figure 4:**
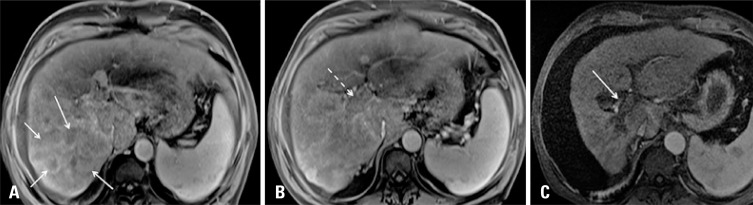
Upper abdomen magnetic resonance, T1-weighted with fat saturation after contrast, in arterial phase, axial section. (A) Expansive formation compatible with infiltrative hepatocellular carcinoma involving most of the right lobe, associated to several satellite lesions (white arrows). B) Observe thrombosis in the portal vein trunk and right branch (dotted white arrow). (C) Upper abdomen magnetic resonance T1-weighted with fat saturation after contrast, in arterial phase, axial section, shows involution of the infiltrative tumor lesion in the right lobe (black arrow) and necrosis of the infiltrative tumor of the portal vein right branch (white arrow). Moreover, observe atrophy of the right lobe (black arrow) and mild paradoxical hypertrophy of the left hepatic lobe (wide white arrow)

A 71-year-old patient suffering from hepatitis C, liver cirrhosis and advanced hepatocellular carcinoma with portal vein thrombosis, not eligible for transplant or surgical resection. Magnetic resonance imaging showed an expansive formation compatible with infiltrative hepatocellular carcinoma involving nearly all right lobe, several satellite lesions ( Figure 4 A) and thrombosis in the portal vein right branch and trunk ( Figure 4 B). In a multidisciplinary meeting, it was decided to perform SIRT.

Magnetic resonance imaging performed six months after SIRT, displayed marked tumor involution and necrosis of the infiltrative tumor of the portal vein. In this case, two side effects of SIRT were observed: atrophy of the right lobe and mild hypertrophy of the left hepatic lobe (paradoxical hypertrophy) ( Figure 4 C).

## DISCUSSION

SIRT is a therapy performed in few hospitals all over the world and provides findings that are characteristic in MRI after treatment – some of them may raise doubts regarding the therapeutic response. In this report we outline the characteristic images of response to treatment; however they are not common comparing to the literature on other locoregional therapies.

Hyperenhancement around the lesion is one of the findings more often observed in MRI after SIRT, corresponding to the inflammation area of the hepatic parenchyma, related to perfusion disorder, as described in case 1. This finding may be misunderstood as tumor viability if the MRI were performed after chemoembolization control.

The particles administered in SIRT can be taken up in subcentimeter lesions that are not visible yet in the MRI before the procedure, and the control images may show necrotic areas in non-target regions, as reported in case 2.

After SIRT, we often observe an increased volume of the tumor lesions, especially if the control image is performed within the first 30 days, what may lead to diagnostic misinterpretations, as shown in case 3. This paradoxical growth is due to appearance of a necrotic area, beyond the tumor margins seen in the previous MRI, and it does not necessarily correspond to poor response to procedure. Therefore, it is recommended to perform a control image at least 60 days after SIRT, to assess the real response to treatment.

The paradoxical hypertrophy of the contralateral hepatic lobe in the SIRT, as seen in case 4, is a condition observed that mimics hypertrophy of the future remaining liver after embolization of the portal vein. The contralateral hepatic lobe may increase by up to 29%.^(^ ^7^ ^)^

Considering the MRI to control the primary or secondary tumors after SIRT, and to avoid misinterpretation of results, the following imaging findings should be mentioned: necrotic areas in unexpected sites, paradoxical increased tumor volume, paradoxical hypertrophy of the contralateral hepatic lobe and atrophy of the hepatic lobe submitted to SIRT.
